# [Corrigendum] Smoothened antagonist GDC-0449 (Vismodegib) inhibits proliferation and triggers apoptosis in colon cancer cell lines

**DOI:** 10.3892/etm.2025.12885

**Published:** 2025-05-14

**Authors:** Chuanqing Wu, Shaobo Hu, Ji Cheng, Guobin Wang, Kaixiong Tao

Exp Ther Med 13:2529–2536, 2017; DOI: 10.3892/etm.2017.4282

Subsequently to the publication of the above article, an interested reader drew to the authors’ attention that the control western blots shown in Figs. 1C and 4C were strikingly similar (although it was conceivable that these blots may have been included as intended in these figures); however, the blots included in Fig. 1C to show the results of the Gli/Caco-2 experiments were also apparently the same as those shown in Fig. 4C for the Bcl-2/Caco-2 experiments, suggesting that, at least in the latter case, the same data had been used to show the results from experiments performed under different conditions.

After having consulted their original data, the authors realized that these figures had been assembled incorrectly, although, due to the time that has elapsed since this paper was published, they no longer had access to their original data. The authors, however, were able to repeat these experiments, and the revised versions of Figs. 1 and 4, containing replacement data for Figs. 1C and 4C, are shown on the next two pages. Note that the results obtained from the repeated experiments were broadly the same as the original results, and the revised data shown for these figures do not affect the overall conclusions reported in the paper. All the authors agree with the publication of this corrigendum; furthermore, they apologize to the Editor of *Experimental and Therapeutic Medicine* and to the readership for any inconvenience caused.

## Figures and Tables

**Figure 1:**
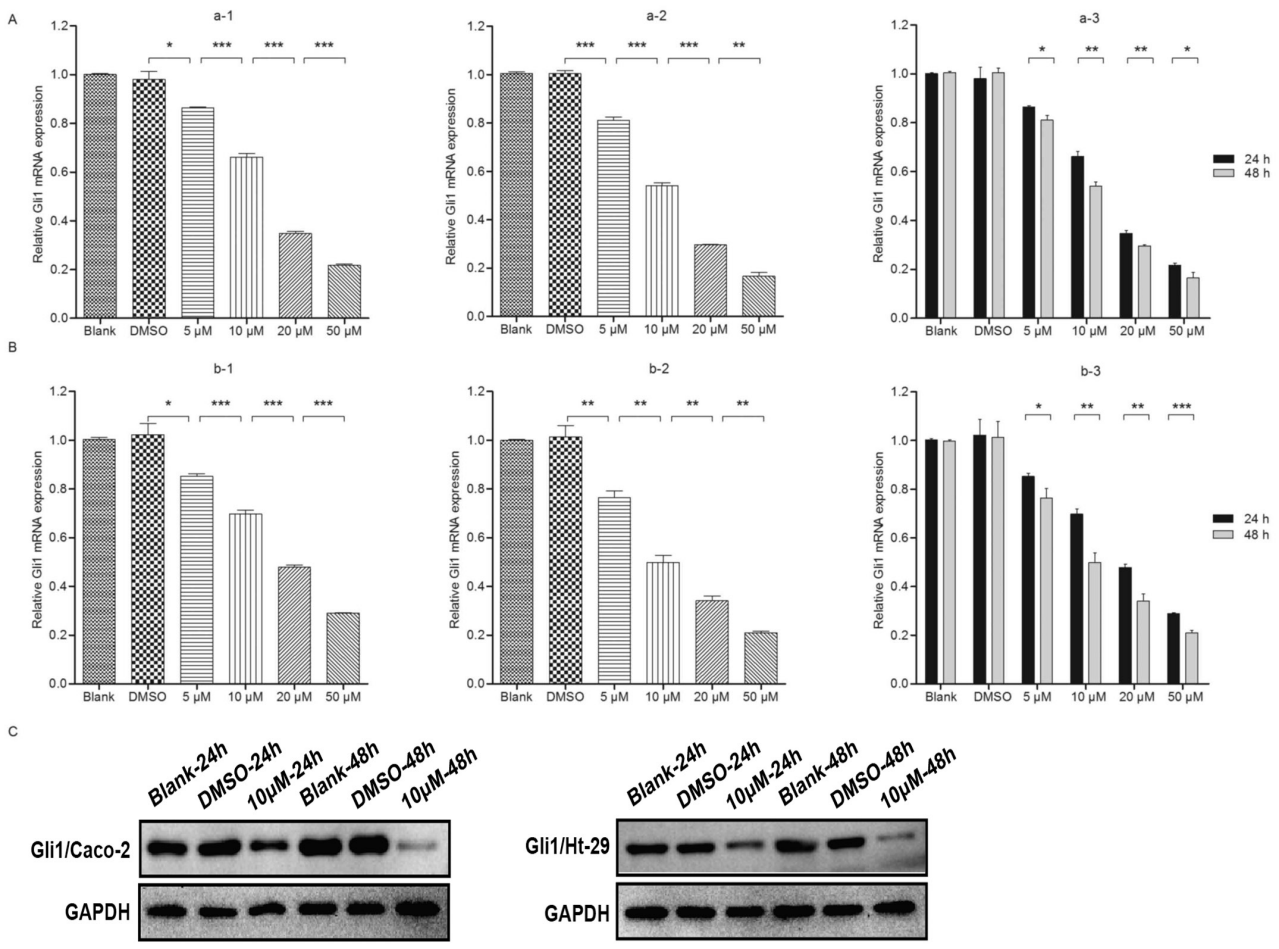
GDC-0449 decreases Gli1 expression in the Caco-2 and Ht-29 cell lines. Gli1 mRNA expression was detected by reverse-transcription quantitative polymerase chain reaction analysis. (A) Caco-2 cell line. Incubation of the blank, DMSO or 5–50 µM GDC-0449 groups for (a-1) 24 h and (a-2) 48 h. (a-3) Comparison of Gli1 mRNA expression in the Caco-2 cell line between 24 and 48 h at various GDC-0449 doses. (B) Ht-29 cell line. Incubation of the blank, DMSO or 5–50 µM GDC-0449 groups for (b-1) 24 h and (b-2) 48 h. (b-3) Comparison of Gli1 mRNA expression in the Ht-29 cell line between 24 and 48 h at various GDC-0449 doses. Values are expressed as the mean ± standard deviation. ^*^P<0.05; ^**^P<0.01; ^***^P<0.001. (C) Western blot analysis of Gli1 protein in Caco-2 and Ht-29 cell lines. In the presence of 10 µM GDC-0449, Gli1 protein expression was obviously decreased compared with the control group, with the effect at 48 h being greater than that at 24 h. Expression levels in the two cell lines were similar. Results are representatives of three independent experiments. DMSO, dimethylsulfoxide.

**Figure 4:**
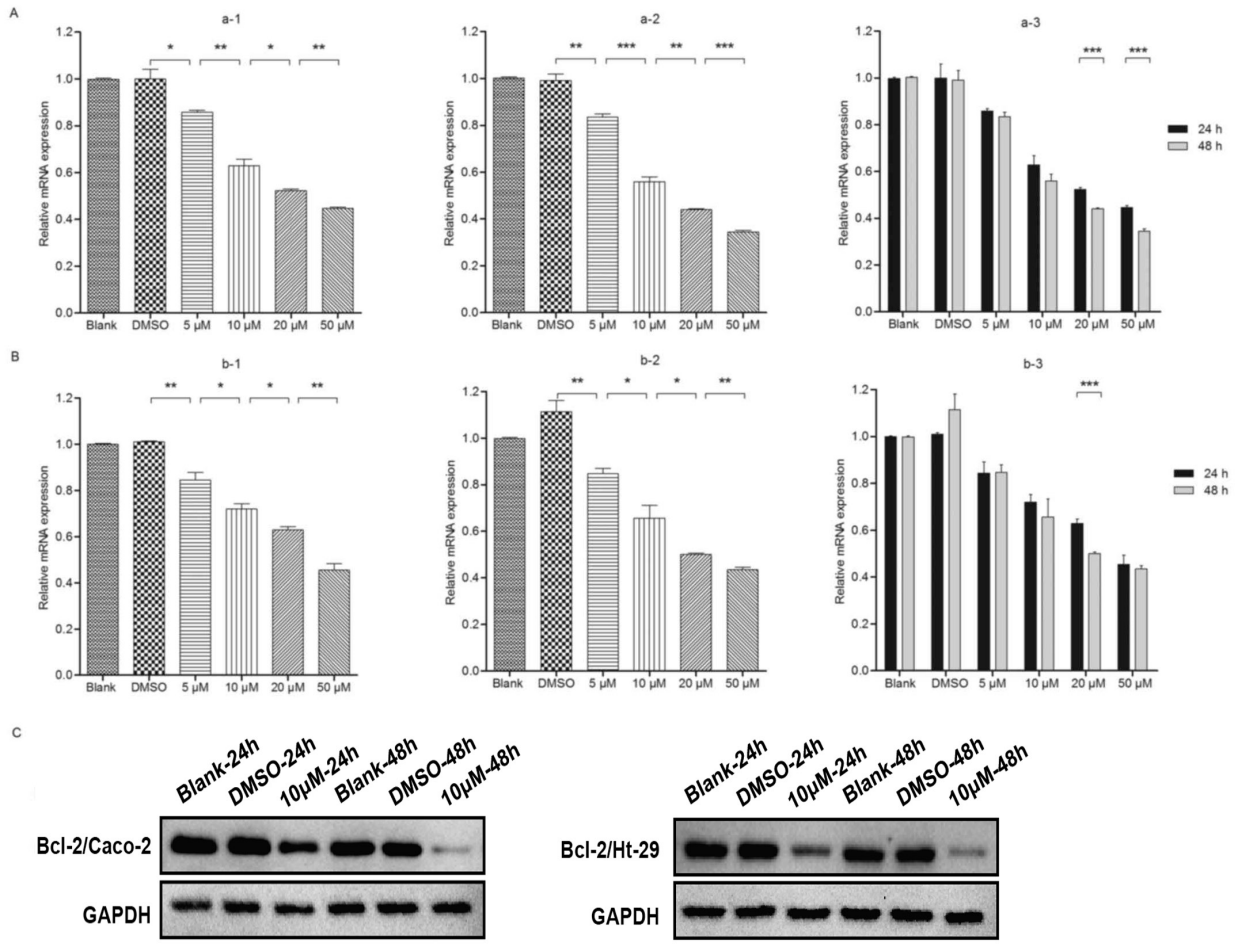
GDC-0449 decreases the expression of Bcl-2 in Caco-2 and Ht-29 cell lines. Reverse-transcription quantitative polymerase chain reaction analysis was used to detect Bcl-2 mRNA. (A) Caco-2 cell line. Blank, DMSO or 5–50 µM GDC-0449 groups incubated for (a-1) 24 h and (a-2) 48 h. (a-3) Comparison of the expression of Gli1 mRNA in the Caco-2 cell line between 24 and 48 h for various GDC-0449 concentrations. (B) Ht-29 cell line. Blank, DMSO or 5–50 µM GDC-0449 groups incubated for (b-1) 24 h and (b-2) 48 h. (b-3) Comparison of the expression of Gli1 mRNA in the Ht-29 cell line between 24 and 48 h for various GDC-0449 concentrations. (C) Western blot analysis of Bcl-2 protein in Caco-2 and Ht-29 cell lines. In the presence of 10 µM GDC-0449, Bcl-2 protein expression was obviously decreased compared with the control group, with the effect at 48 h being greater than that at 24 h. The two cell lines showed similar results. Results were determined from three independent experiments. Values are expressed as the mean ± standard deviation. ^*^P<0.05; ^**^P<0.01; ^***^P<0.001. DMSO, dimethylsulfoxide; Bcl-2, B-cell lymphoma 2.

